# Small Molecule Follicle-Stimulating Hormone Receptor Agonists and Antagonists

**DOI:** 10.3389/fendo.2018.00757

**Published:** 2019-01-23

**Authors:** Ross C. Anderson, Claire L. Newton, Robert P. Millar

**Affiliations:** ^1^Centre for Neuroendocrinology, Department of Physiology, Faculty of Health Sciences, University of Pretoria, Pretoria, South Africa; ^2^Centre for Neuroendocrinology, Department of Immunology, Faculty of Health Sciences, University of Pretoria, Pretoria, South Africa; ^3^Institute of Infectious Disease and Molecular Medicine, Department of Integrative Biomedical Sciences, Faculty of Health Sciences, University of Cape Town, Cape Town, South Africa

**Keywords:** follicle-stimulating hormone (FSH), FSH receptor (FSHR), agonists, antagonists, small-molecule, GPCR (G protein-coupled receptors), assisted reproduction (ART)

## Abstract

The follicle-stimulating hormone receptor (FSHR) has been targeted therapeutically for decades, due to its pivotal role in reproduction. To date, only purified and recombinant/biosimilar FSH have been used to target FSHR in assisted reproduction, with the exception of corifollitropin alfa; a modified gonadotropin in which the FSH beta subunit is joined to the C-terminal peptide of the human choriogonadotropin beta subunit, to extend serum half-life. Assisted reproduction protocols usually entail the trauma of multiple injections of FSH to initiate and promote folliculogenesis, which has prompted the development of a number of orally-available low molecular weight (LMW) chemical scaffolds targeting the FSHR. Furthermore, the recently documented roles of the FSHR in diverse extragonadal tissues, including cancer, fat metabolism, and bone density regulation, has highlighted the potential utility of LMW modulators of FSHR activity. Despite these chemical scaffolds encompassing a spectrum of *in vitro* and *in vivo* activities and pharmacological profiles, none have yet reached the clinic. In this review we discuss the major chemical classes of LMW molecules targeting the FSHR, and document their activity profiles and current status of development, in addition to discussing potential clinical applications.

## Introduction

The hypothalamic-pituitary-gonadal axis comprises hypothalamic kisspeptin and neurokinin B (NKB) driving the secretion of gonadotropin-releasing hormone (GnRH). GnRH subsequently stimulates the pituitary secretion of the gonadotropin hormones luteinizing hormone (LH) and follicle stimulating hormone (FSH), into the general circulation, resulting in gonadal steroidogenesis, and pubertal development via activation of their cognate gonadotropin receptors, FSH receptor (FSHR), and LH receptor (LHR/LHCGR). In this article we review the development and potential clinical application of small molecule/low molecular weight (LMW) modulators of FSHR activity.

The FSHR is a G protein-coupled receptor (GPCR) that belongs to the glycoprotein hormone receptor sub-family of GPCRs that also includes the luteinizing hormone receptor (LHR/LHCGR), and the thyroid-stimulating hormone receptor (TSHR). These GPCRs are characterized by the presence of large extracellular N-terminal ectodomains (ECDs) that bind the heterodimeric glycoprotein hormones, in addition to the classical seven transmembrane domain region (TMD) characteristic of the GPCR superfamily. FSHR predominantly couples to and activates the Gα_s_ class of intracellular G proteins, resulting in adenylyl cyclase stimulation, and a subsequent increase in the second messenger cyclic adenosine monophosphate (cAMP). cAMP then binds to and modulates the activity of a number of cyclic nucleotide-binding proteins, including cAMP-dependent protein kinases, and ion channels.

While Gα_s_ is considered the main effector of FSHR-mediated signaling, Gα_q_-mediated signaling, and β-arrestin mediated (G protein-independent) signaling have also been observed (1–3). These different signaling modalities are responsible for the activation of a multitude of downstream effectors, thus representing a complex network of possible signaling outcomes (2).

It is necessary that the full complement of possible signaling pathways is acknowledged both in the context of gonadal steroidogenesis, but also drug development, as a number of LMW molecules (described in detail below) are emerging with selective signaling profiles (a phenomena referred to as “biased-signaling”). These molecules have greatly informed as to the pathways involved (and required) for successful gonadal steroidogenesis, while simultaneously highlighting the inherent dangers of *in vitro* characterization of LMW molecules targeting the FSHR by measuring cAMP response in isolation.

Despite the successful application of corifollitropin, which comprises a hybrid molecule in which the FSHβ subunit is fused to the C-terminal 24 amino acids of the human chorionic gonadotropin β subunit (hCGβ) to increase serum half-life (marketed under the trade name Elonva) in assisted reproduction (4) and taking into account the signaling complexities discussed above there still remains a drive to develop LMW modulators of the gonadotropin receptors. The utilization of LMW orally-active modulators of FSHR has many theoretical advantages. Multiple injections (and associated site irritation) of polypeptide FSH during assisted reproduction would be avoided. Such classical LMW orally-active pharmaceutical compounds are also potentially superior in their greater stability and uniformity unlike gonadotropin polypeptides which require refrigeration and are subject to variable post-translational glycosylation which might affect half-life and bioactivity (5–8). The desirable properties of LMW analogs would therefore potentially result in more clinically effective treatment regimens. Another advantage of using orally-active gonadotropin analogs is the potential to vary dose which may have an additional benefit in avoiding the vexing and potentially life-threatening condition of ovarian hyperstimulation syndrome (OHSS).

In addition to these potential advantages, orally bioavailable LMW FSH antagonists may have potential as oral contraceptives. Current sex steroid-based contraceptives are administered at supra physiological doses to inhibit ovulation which can increase the risk of side effects, such as cardiovascular thrombosis events associated with estradiol-based contraceptives (9). It is arguable that some of these side effects would be mitigated by targeting the FSHR, although the potential health risks of increased pituitary FSH release in response to antagonism of FSHR would require investigation. This is in light of the reported links between FSH oversecretion and the progression of certain cancers, bone loss, and increased body fat (10, 11), although it might be predicted that at least some of these effects would be mitigated by the presence of the FSHR antagonist (see *Concluding remarks and future perspectives*).

Despite the theoretical therapeutic potential of LMW modulators of FSHR, a number of substantial challenges needed to be overcome. The FSHR is a leucine-rich repeat containing GPCR, belonging to the glycoprotein hormone receptor family, which also includes the TSHR, and LHR. In addition, there are other GPCRs containing leucine-rich-repeat motifs. These GPCRs share high degrees of sequence conservation with the FSHR suggesting that drug cross-reactivity/specificity could be a potential problem. Moreover, the gonadotropins are large dimeric proteins that contact the gonadotropin receptors via multiple residues that include the glycan moieties, in addition to having a complex mechanism of receptor activation that includes structural movements within the extracellular domain (ECD) and the transmembrane (TM) domains of the receptor. As a result, it appeared that a LMW molecule might not fulfill the requirements of both receptor binding and activation. Nevertheless, these challenges could be successfully addressed in the development of both LHR and FSHR orthosteric agonists and antagonists (whose binding site overlaps with that of the natural ligand), and allosteric analogs which interact with the receptor at a site distinct from the orthosteric ligand binding site (12).

Allosteric GPCR modulators can be categorized based on measures of gonadotropin receptor signaling activity (most frequently measurement of cAMP, but biased modulators have also been described), and fall into three groups; allosteric agonists (allo-agonists) and positive and negative allosteric modulators (PAMs and NAMs). Allosteric agonists have agonist activity, in the absence of gonadotropin, in contrast to PAMs and NAMs whose activity can only be demonstrated in the presence of agonist, usually via modulation of orthosteric hormone binding affinity or altering the ability of the receptor to interact with intracellular signal transducers. In this way, PAMs and NAMs can either augment or diminish the response to orthosteric agonists, respectively. PAMs and NAMs have garnered much interest in the pharmaceutical industry, as these classes of allosteric modulators only have activity in the presence of co-bound endogenous ligand meaning that GPCR activation is limited to the spatio-temporal release of endogenous ligands. This is extremely advantageous, as many of the reproductive neuroendocrine hormones are released/secreted in cyclical patterns or pulses (13).

**Figure 1:**
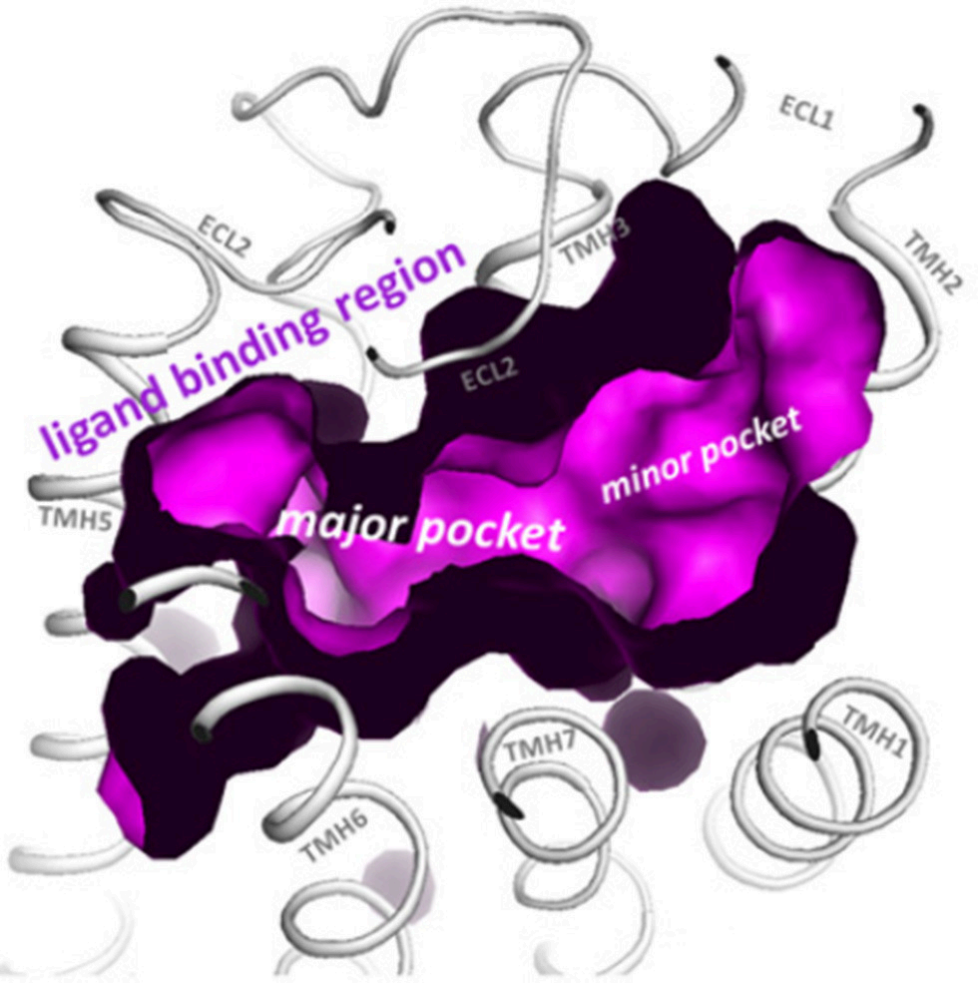
The top view of the LHR with partially removed extracellular loops (ECLs) highlights the two putative allosteric binding sites for LMW gonadotropin receptor ligands (magenta), located within the transmembrane (TM) domains and adjacent to the ECLs. These sites are labeled the P1 (major) site and P2 (minor) site. Reproduced with permission from (10).

Structural modeling has been successfully utilized to identify allosteric sites in GPCRs for LMW compounds. Gonadotropin receptor TM domains have been modeled using adenosine receptor crystal structures, and identified two putative allosteric sites within the TM domains, positioned adjacent to the ECL loops (14, 15). These putative TM domain allosteric sites have been confirmed in studies utilizing chimeric trophic hormone receptors, and mutagenesis approaches. These sites have been assigned P1 and P2 (major site and minor site respectively) (14, 16, 17) (Figure 1). The P1 site is located between TMs III, IV, V, and VI, and P2 between TMs I, II, III, and VII (15).

Several cell-based screening assays have successfully been employed to identify structural scaffolds that can allosterically bind to the gonadotropin receptors (18). The large array of chemical scaffolds with FSHR activity have revealed a number of interesting and unique activity profiles, both *in vitro* and *in vivo*, with allo-agonists, NAMs, and PAMs identified. While LMW compounds targeting the closely-related, LHR have been identified, most endeavor has been directed toward developing LMW molecules targeting the FSHR. This is predictable, given that ovarian hyperstimulation requires multiple injections of FSH, in contrast to the single administration of LH/hCG (or other stimulus, such as GnRH agonist) needed to induce ovulation. Despite considerable progress in the development of LMW FSH analogs, none have yet entered the clinic. In many cases, this is a result of *in vitro* activity failing to translate into *in vivo* activity, off-target effects/toxicity, synthesis issues, and frequently termination of research programs following pharmaceutical company acquisitions, and differing priorities of the acquiring company. These issues have been previously reviewed in detail (18). Here we review the progress that has been made in developing LMW orally active FSHR analogs, and discuss their potential clinical applications.

## FSHR Agonists

### Thiazolidinones

**Figure 2:**
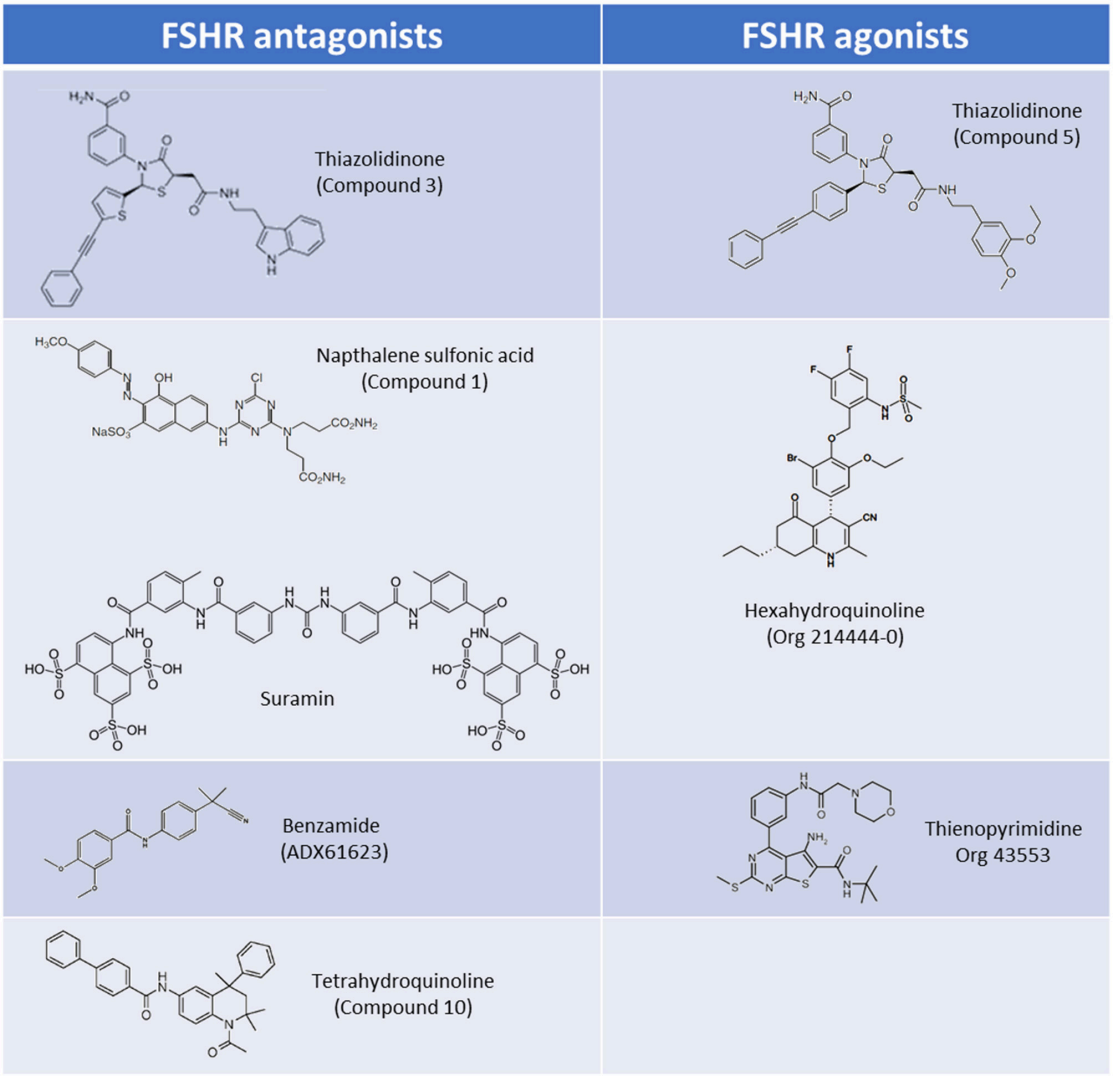
Core scaffolds for the major classes of LMW FSHR agonists and antagonists.

Thiazolidinone (TZD) core scaffolds have previously been utilized in a number of successful GPCR drug discovery programs, and offer a flexible and versatile platform for compound development (19). Affymax (Cupertino, US) utilized a TZD compound library containing 42,000 molecules, and identified several lead compounds with agonist activity at the FSHR. Several hits with nanomolar (nM) potencies were identified from this screen including a partial agonist (E_max_ 24% of maximal recombinant FSH stimulation) with an EC_50_ of 32 nM (20). This lead compound was subsequently modified by adding γ-lactam congeners and 5-alkyl substituents in an attempt to address stereoselectivity issues with the heterocyclic ring in which the *trans* isomer predominated over the active *cis* isomer during synthesis (21, 22). A parallel drug discovery program run between Affymax and Wyeth [since acquired by Pfizer (New York City, US)] also screened a large compound library representing a spectrum of core scaffolds, with two of the best hits containing TZD core structures. These lead compounds had poor EC_50_'s of approximately 20 μM, but following parallel synthesis resulted in three promising compounds with nanomolar potencies and full efficacy *in vitro* (1–6 nM) (23). Subsequent studies in which chimeric receptors were created via exchanging of the N-termini and TM domains of the TSHR and FSHR supported the notion that that the analogs bind within the TM domain of the FSHR. The site of interaction of these allo-agonists with the FSHR was further refined to a site located between TM I and ECL2 (23). A compound, (compound 5) (Figure 2), was identified and was demonstrated to be capable of stimulating steroid production in FSHR transfected rat primary granulosa cells and mouse adrenal Y1 cells to the same level as FSH, albeit with approximately 1,000-fold lower potency (23). *In vivo* activity was evaluated in a rat ovulation assay, where a dose-dependent increase in the number of ovulated oocytes was observed, however, poor oral bioavailability, and genotoxic effects were noted stalling further development (24).

The pharmacological properties of TZD-containing compounds was further evaluated, and intriguingly it was discovered that minor modification to the core thiazolidine ring, at either an aryl group at position 3 or an acetic acid amide chain at position 5 of the thiazolidine ring resulted in differing pharmacological profiles to the parent compound (25). These activities ranged from agonists (activating Gα_s_) through to NAMs which inhibited estradiol production in rat granulosa cells via Gα_i_ activation (“compound 3”; Figure 2) (25). These simple TZD core structures therefore have the potential to deliver a spectrum of LMW allosteric modulators targeting multiple FSHR signaling pathways, should synthesis, bioavailability, and toxicity issues be mitigated.

### Diketopiperazines

Pharmacopeia Inc. (now Ligand Pharmaceuticals, San Diego, United States) and Organon [now Merck & Co/Merck Sharpe & Dohme (MSD), Kenilworth, United States] screened a large diketopiperazine compound library. A number of biaryl agonists were identified, with the most potent compounds containing heterocyclic diketopiperazine substituents (26). Lead optimisation, through modification of the diketopiperazine core side chains, led to increased potency of activation, (in the nanomolar range) in a cAMP-response element (CRE)-containing luciferase reporter gene assay, and a cAMP accumulation assay (27). These compounds were apparently not developed further and there is no information on specificity or *in vivo* activity. Interestingly, other piperazine compounds with FSHR activity have been described, and a patent was submitted by Serono, describing low nanomolar FSHR piperazine agonists (28).

### Hexahydroquinolines

Poor oral activity of many LMW compounds has hampered development. Organon demonstrated the first high potency LMW compound with good oral activity targeting the FSHR following the filing of patents on the use of hexahydroquinoline scaffolds (4-phenyl-5-oxo-l,4,5,6,7,8-hexahydroquinolines) as FSHR activators (29, 30). These LMW compounds had potencies of < 10 nM in an *in vitro* CRE-luciferase reporter gene assay, and subsequently a hexahydroquinoline LMW allosteric agonist at the FSHR, Org 214444-0 was described (31) (Figure 2). Co-incubation with FSH resulted in a substantial increase in FSH affinity and FSH potency in the CRE-luciferase reporter gene assay, confirming that this compound was an FSHR PAM (31). Administration of Org 214444-0 induced follicular growth in a rat ovulation assay following oral dosing at 1 mg/kg every 4 h in mature cycling rats, by potentiating endogenous FSH activity, thus demonstrating oral-activity of an FSHR LMW agonist for the first time (31).

### Thienopyrimidines

Organon reported a number of orally-active thienopyrimidine and thienopyridine compounds with activity at the LHR, both *in vitro* and *in vivo* (32–34). Lead compound optimisation resulted in a thienopyrimidine compound (Org 43553; Figure 2), with low nanomolar potency at the LHR, but also activity at the FSHR (approximately 10-fold reduced potency vs. LHR). Radioligand dissociation assays and the generation of chimeric LHR/TSHR receptors showed that Org 43553 interacts with a single allosteric site, located in the LHR TM domains (35, 36). Interestingly, Org 43553 appeared to induce an active conformation of the receptor necessary for adenylyl cyclase activation, but not inositol phosphate (IP) generation, unlike the endogenous hormones (36). Furthermore, Org 43553 was demonstrated to inhibit LH-induced IP production (36). Following successful induction of ovulation in rodents (37) this compound was tested for safety and efficacy in humans following oral administration in a pre-clinical trial, where it was tolerated in doses up to 2,700 mg, and induced ovulation in healthy females (38). Interestingly, selectivity of Org 43553 for LHR was improved via linkage of two Org43553 molecules, with flexible or rigid linkers, or by conjugating an FSHR LMW antagonist, implying that modification to abolish gonadotropin receptor cross-reactivity is possible (39, 40). The thienopyrimidine class of compounds therefore represent a promising scaffold from which to develop LMW modulators of FSHR.

### Agonists With Undisclosed Scaffolds/Other Scaffolds

Piperidine carboxyamide derivatives were identified in a high-throughput screen by Serono (now Merck KGaA, Darmstadt, Germany). Lead compounds were shown to have low nanomolar potency *in vitro* in a luciferase reporter gene assay, but poor potency in an estradiol production assay utilizing rat granulosa cells (41).

MSD identified two LMW FSHR agonists with undisclosed scaffolds. These agonists had low nanomolar potency at the FSHR, but were notable for their unusual very short-acting profiles with a half-life and T_max_ of approximately 1.5 and 0.5 h respectively) compared to recombinant FSH [half-life approximately 24–48 h (42–44)]. These compounds were subsequently shown to inhibit ovulation, and induce the production of luteinized unruptured follicles in cycling rats when administered orally, with complete reversal of the effect, and resumption of cyclicity following withdrawal of the compounds (44). These compounds may yet form the basis for an effective novel contraceptive, but the inability to reproduce a similar effect in primates has limited their immediate therapeutic potential (44). The mechanism was apparently not investigated and possibly resulted from desensitization of the FSHR.

## FSHR Antagonists

### Sulfonic Acid

Suramin, a sulfonic acid-containing compound (Figure 2), is an established treatment for trypanosomiasis (African sleeping sickness) and has been used for almost a century (45). Suramin has been shown to inhibit the signaling of a number of peptide hormones/receptors. Interestingly, rats treated with suramin displayed decreased plasma testosterone and FSH levels, and *in vitro* experiments showed suramin-mediated inhibition of both hCG and FSH activities (46). Subsequent radioligand competition-binding experiments have suggested that suramin interacts with the orthosteric ligand binding pocket of FSHR (46, 47). It has also been suggested that suramin inhibits ternary complex formation, by blocking GPCR-G protein interactions, preventing GDP-release (48, 49), but the validity of these claims remains to be established, in light of the fact that suramin has been demonstrated to inhibit receptor tyrosine kinases, such as epidermal growth factor receptor, and platelet-derived growth factor receptor, which do not couple to G proteins (45). Suramin has also been tested in patients with refractory cancers including prostate cancer. In these patients decreases in plasma testosterone, FSH and prostate specific antigen have been observed (50, 51), and while overall survival rates were unchanged between placebo and suramin treatment groups, there were suggested palliative benefits with reduced pain, and opioid analgesic uptake (51). These observations have led to increased interest in suramin within the arena of GPCR drug development, and despite poor oral uptake (suramin is administered intravenously), have propagated interest in sulfonic acid containing LMW compounds as possible modulators of FSHR activity.

### (Bis)Sulfonic Acid, (Bis)Benzamides

Three (bis)sulfonic acid, (bis)benzamide FSHR antagonists were identified, with moderate potencies of activation in a number of *in vitro* assays (52). While suramin appears to interact with a number of GPCRs, the (bis)sulfonic acid, (bis)benzamide FSHR LMW antagonists displayed no LHR and TSHR binding and signaling activity up to 100 μM (52). Concurrently, Wyeth identified a naphthalene sulfonic acid compound (“compound 1”; Figure 2) that non-competitively inhibited FSH binding to the FSHR, despite binding to the FSHR extracellular domain (ECD), resulting in a reduction of FSH binding sites but not affinity (53). In addition this compound completely abolished the cAMP response to FSH (53). This was substantiated *in vivo* in cycling female rats receiving 100 mg/kg i.p. which inhibited ovulation in all treated animals (53). Issues with oral bioavailability, and safety/off-target effects, including mild inflammation of the ovarian surface epithelium and growth retardation have hindered the successful development and transition of these compounds into the clinic despite promising *in vitro* activity profiles (53). Modification to improve oral absorption of these compounds was attempted, but carboxylic acid substituents abolished FSHR binding activity (52).

### Tetrahydroquinolines

A tetrahydroquinoline (THQ) scaffold containing an amino group at position 6 with modest micromolar FSHR agonistic activity and an efficacy of 85% relative to FSH, was identified by Organon using a CRE-luciferase reporter assay. In a similar vein to the TZD agonists, minor modification of the core structure, in this case introduction of an aromatic group at position 6 of the THQ scaffold, resulted in compounds with completely different pharmacologies. Aromatic groups incorporated at position 6 resulted in a switch from full agonists to full antagonists, with nanomolar IC_50_'s (compound 10; Figure 2) (54). It was established that the binding pocket was likely large and lipophilic, given that large aromatic substituents at position 6 of the THQ scaffold were tolerated (including biphenyl groups), also suggesting that the compounds likely bound in the TM domain. In support of this, competition binding assays showed lack of displacement of FSH binding by these compounds (54). Additionally, it was also shown that these aromatic groups were preferred for antagonistic activity (54). In a mouse *ex vivo* follicular growth assay one of the biphenyl-substituted THQ compounds inhibited follicular growth in the presence of FSH, and substantially inhibited ovulation (up to 78% of follicles) (54).

### Benzamide Derivatives

Addex Pharmaceuticals (Geneva, Switzerland) used a homogenous time resolved fluorescence (HTRF) screening assay to screen for FSHR NAMs, and identified a benzamide compound (ADX61623; Figure 2) with activity (IC_50_ 0.7 μM with 55% inhibition of FSH EC_80_). Interestingly, ADX61623 inhibited cAMP and progesterone production in the presence of FSH *in vitro* in a dose-dependent manner, and conversely stimulated estradiol production at high concentrations (55). These results suggest that cAMP signaling is not a requirement for estradiol production. Indeed, it is known that the ovarian functions of FSH (such as estradiol production) are under the control of a number of signaling pathways (see *Introduction*). In an *in vivo* setting, administration of 50 mg/kg ADX61623 s.c. was ineffective in completely inhibiting folliculogenesis and ovulation in rats following sequential treatment with pituitary FSH and hCG, as measured by number of oocytes recovered and ovarian weight (55). The authors postulate that this may be due to the inability of ADX61623 to inhibit the production of estradiol despite inhibition of cAMP. Two additional benzamide analogs, identified by Addex, (ADX68692 and ADX68693) corroborated the antagonistic activity profile of ADX61623, substantiating the requirement for blockade of both arms of the FSHR steroidogenic signaling pathway to inhibit follicular maturation and ovulation in rats (56). Indeed, while subcutaneous or oral administration of ADX68692 (which was demonstrated in primary rat granulosa cells to inhibit progesterone and estradiol production) was shown to disrupt cyclicity in mature female rats, and reduced the number of oocytes recovered, ADX68693 which inhibited progesterone but not estradiol production, had no effect (56). Interestingly, ADX68692 and ADX68693 demonstrated biased-activity profiles at the LHR as well as the FSHR, with ADX68693 abolishing testosterone and inhibiting progesterone production in rat primary leydig cells while ADX68692 partially inhibited testosterone and potentiated progesterone production (57). Many LMW compound screening strategies assay for a single signaling output, with the benzamide compound series highlighting the inherent dangers of this strategy, and may in part explain the failure of many gonadotropin LMW compounds to translate promising *in vitro* efficacy to *in vivo* bioactivity.

### Other FSHR Antagonists

In addition to the compound families described above, acyltryptophanol and pyrrolobenzodiazepine LMW FSHR antagonists have been identified through HTRF and CRE-luciferase screening assays respectively, with a spectrum of IC_50_ concentrations (58, 59). The current status of these programs is unknown.

Additionally, a novel substituted aminoalkylamide series of FSHR modulators have been identified with antagonistic activity. Ortho-McNeil Pharmaceutical discovered two potent compounds (identified in a cAMP screening assay) but they failed to effectively inhibit ovulation and spermatogenesis in Wistar rats (60). To determine whether the inability of the compounds to inhibit FSHR *in vivo* was due to restricted oral bioavailability, poor intestinal absorption, and/or rapid clearance, additional pharmacokinetic data would be required.

## Concluding Remarks and Future Perspectives

Natural and recombinant gonadotropins have been the mainstay of infertility treatment in men and women for decades, and are still an essential component of assisted reproductive technologies which have impressively addressed the needs of infertile patients world-wide. However, there is a perceived need to develop gonadotropin analogs and orally-available LMW compounds with gonadotropin receptor activity which would obviate current multiple injection protocols, and importantly, reduce OHSS risk. Modulation of relative FSH and LH activities would also have application in women's health conditions such as polycystic ovarian syndrome (PCOS) and would possibly offer a novel method of contraception. Other potential applications of gonadotropin receptor modulators could include the management of animal reproduction, both as contraceptives in population control, and utilization in assisted reproduction for animal husbandry purposes and in endangered animals. With regards to the latter, IVF and cryopreservation protocols have been applied with varying degrees of success in endangered ungulates, and felid species (61–63).

Although several promising FSHR LMW agonists and antagonists have been shown to have desirable properties in animal models, none have shown efficacy in human clinical studies. This is in contrast to LHR LMW molecules which have been demonstrated to be efficacious in stimulating ovulation in women. One example is MK-8389 (developed by MSD) which had promising activity in a rat model but when tested in an ascending dose study on pituitary-suppressed females, showed an effect on thyroid function, despite no effect on follicular development, or estradiol production (64). Thus, further research endeavors are required to produce efficacious orally-active FSH LMW agonists as substitutes for multiple injections of conventional polypeptide FSH.

Despite these setbacks, the pursuit of FSHR LMW compounds with *in vitro* activity has been highly successful with the impressive development of dozens of allosteric compounds with appropriate properties. The majority of these LMW compounds have arisen from a relatively limited number of core scaffolds, each with distinctive chemistries, and an array of interesting properties. Amongst these allosteric compounds are NAMs and PAMs which have unique properties for exploitation, such as biased-activity profiles, which may be of value in differential stimulation or inhibition of estradiol and progesterone. In view of the diverse structures of molecular scaffolds utilized it is highly likely that following sufficient investment and development, several of these would have the appropriate characteristics and safety profile to be therapeutics.

The acquisition of reproductive health companies by big pharma may have played a significant role in the termination of LMW gonadotropin analog development, due to differing research priorities. For example, the acquisition of Wyeth by Pfizer may have halted development of the TZD FSHR agonist. Encouragingly, a number of small biotech companies are still focussing on the development of LMW modulators of FSHR activity, and we may yet see compounds entering the clinic for a diversity of applications.

An exciting new development in the arena of LMW gonadotropin analogs is the discovery that some of these compounds have gonadotropin receptor “pharmacological chaperone” or “pharmacoperone” activity. These pharmacoperone compounds can restore plasma membrane localization and function to intracellularly-retained GPCRs harboring mutations that result in misfolding and intracellular retention/degradation. LMW LHR allosteric agonists have been shown to act as pharmacoperones, “rescuing” cell surface expression of intracellularly retained mutant LHRs (65) (an outcome of the majority of inactivating point mutations in the LHR) (66). We and others have demonstrated that at high concentrations the LHR LMW molecules are also able to restore cell surface expression to intracellularly retained human FSHR mutants (67). As there is currently no treatment for patients harboring these mutations, the LMW pharmacoperones potentially represent an exciting novel breakthrough in personalized and precision medicine in reproduction.

It has been suggested that the FSHR is expressed in a number of extragonadal tissues, and current research implies that these receptors may be physiologically important. Indeed, the utilization of LMW gonadotropin analogs targeting the FSHR may extend beyond the current remit of assisted reproduction and may yet herald a new era of gonadotropic therapeutics (68, 69). For example, post-menopausal women have low oestrogens, and elevated FSH, with concomitant bone loss, and increased body fat. It has been implied that FSH as well as low oestrogens may be playing a role, and indeed in ovariectomised mice, an FSHβ neutralizing antibody was found to reduce bone resorption and stimulate bone synthesis (10). In a subsequent publication the neutralizing antibody was demonstrated to induce thermogenic adipose tissue and additionally reduced body fat and increased the lean mass/total mass ratio, compared to control IgG (11). There are, however, opposing views, on the physiological relevance of extragonadal FSHR, and also contradictory findings in *in vivo* studies. The intimate relationship between FSH/FSHR and estradiol frequently confuse and confound the interpretation of data, in addition to the complicated often-opposed effects of the two hormones such as on osteoclasts (and the additional roles of activins in bone). Another area of potential FSHR LMW analog application is in oncology. Reported proliferative effects of FSH and FSHR in prostatic and ovarian cancers alludes to other novel applications for FSHR LMW agonists and antagonists (70–73), although as with other putative extragonadal functions of FSH/FSHR these data are frequently contradictory. Thus, more research is required to fully understand the physiological mechanisms behind these phenomena, and exploit possible therapeutic opportunities.

## Author Contributions

All authors listed have made a substantial, direct and intellectual contribution to the work, and approved it for publication.

### Conflict of Interest Statement

RM undertakes consultancy work for company KaNDy Therapeutics Ltd. The remaining authors declare that the research was conducted in the absence of any commercial or financial relationships that could be construed as a potential conflict of interest.
